# Age-Friendly as Tranquilo Ambiente: How Socio-Cultural Perspectives Shape Older Latinos’ Lived Environment

**DOI:** 10.1093/geroni/igab046.911

**Published:** 2021-12-17

**Authors:** Melanie Plasencia

**Affiliations:** University of California, Berkeley, Albany, California, United States

## Abstract

Researchers have increasingly considered the importance of age-friendly communities to improve the health and well-being of older adults. Studies have primarily focused on the built environment, such as community infrastructure, older adult behavior, and environmental expectations. Less attention, however, has been given to the role of cultural characteristics in shaping perceptions of age-friendly environments for Latinos. Using an ethnographic methodological approach, including participant observation in a Latino community near New York City and 72 semi-structured interviews, this study provides empirical insights into how older Latinos characterize age-friendly communities. Latino older adults described their community as age-friendly using Tranquilo Ambiente (TA), which translates to a calm or peaceful environment. According to older adults, a TA possesses the following: 1) a sense of personal safety, including protection of their body, 2) ethnic, social connectedness, including networks with other Latinos and important social and cultural events; and 3) a comparative understanding of their communities treatment of seniors versus other geographical and spatial locations. While much has been written on the role of the built and social environment in developing and implementing age-friendly communities, more research on the cultural significance and understanding of place among marginalized older adults is necessary. TA and its characteristics demonstrate that cultivating an age-friendly setting requires adapting structures and services to promote Latino older adults' social and cultural support and engagement.

